# Does the Mediterranean Diet Have Any Effect on Lipid Profile, Central Obesity and Liver Enzymes in Non-Alcoholic Fatty Liver Disease (NAFLD) Subjects? A Systematic Review and Meta-Analysis of Randomized Control Trials

**DOI:** 10.3390/nu15102250

**Published:** 2023-05-09

**Authors:** Cristian Del Bo’, Simone Perna, Sabika Allehdan, Ayesha Rafique, Sara Saad, Fahad AlGhareeb, Mariangela Rondanelli, Reema F. Tayyem, Mirko Marino, Daniela Martini, Patrizia Riso

**Affiliations:** 1 Department of Food, Environmental and Nutritional Sciences (DeFENS), Division of Human Nutrition, Università degli Studi di Milano, 20133 Milano, Italymirko.marino@unimi.it (M.M.);; 2 Department of Biology, College of Science, University of Bahrain, Sakhir Campus, Zallaq P.O. Box 32038, Bahrain; sallehdan@uob.edu.bh (S.A.); ayesha.rafique121@hotmail.com (A.R.); helal.sara95@gmail.com (S.S.);; 3 IRCCS Mondino Foundation, 27100 Pavia, Italy; mariangela.rondanelli@unipv.it; 4 Unit of Human and Clinical Nutrition, Department of Public Health, Experimental and Forensic Medicine, University of Pavia, 27100 Pavia, Italy; 5 Department of Human Nutrition, College of Health Sciences, QU Health, Qatar University, Doha P.O. Box 2713, Qatar; reema.tayyem@qu.edu.qa

**Keywords:** Mediterranean diet, nonalcoholic fatty liver disease, lipid profile, liver function, human interventions

## Abstract

The effectiveness of the Mediterranean diet (MD) in non-alcoholic fatty liver disease (NAFLD) subjects has been evaluated in several randomized controlled trials (RCTs). This systematic review and meta-analysis aimed to evaluate the overall effects of MD intervention in a cohort of NAFLD patients targeting specific markers such as central obesity, lipid profile, liver enzymes and fibrosis, and intrahepatic fat (IHF). Google Scholar, PubMed, and Scopus were explored to collect relevant studies from the last 10 years. RCTs with NAFLD subjects were included in this systematic review with a mean intervention duration from 6 weeks to 1 year, and different intervention strategies, mainly including energy restriction MD (normal or low glycaemic index), low-fat MD with increased monounsaturated and polyunsaturated fatty acids, and increased exercise expenditure. The outcomes measured in this meta-analysis were gamma-glutamyl transferase (GGT), alanine aminotransferase (ALT), total cholesterol (TC), waist circumference (WC), and liver fibrosis. Ten randomized controlled trials, which involved a total of 737 adults with NAFLD, were included. According to the results, the MD seems to decrease the liver stiffness (kPa) by –0.42 (CI95% –0.92, 0.09) (*p* = 0.10) and significantly reduce the TC by –0.46 mg/dl (CI95% –0.55, −0.38) (*p* = 0.001), while no significant findings were documented for liver enzymes and WC among patients with NAFLD. In conclusion, the MD might reduce indirect and direct outcomes linked with NAFLD severity, such as TC, liver fibrosis, and WC, although it is important to consider the variations across trials. Further RCTs are necessary to corroborate the findings obtained and provide further evidence on the role of the MD in the modulation of other disorders related to NAFLD.

## 1. Introduction

Non-alcoholic fatty liver disease (NAFLD) is an umbrella term, under which lies a range of conditions [1]. NAFLD is characterized by the accumulation of fat in the liver, is more prevalent among overweight and obese individuals, and is caused by factors other than excessive alcohol consumption [2]. Among patients with chronic liver diseases, 45.8% were reported to be caused by NAFLD compared to 81.8% by alcoholic liver disease (ALD) [3]. It is also reported to affect about 25% of the world’s population [4].

NAFLD is diagnosed by imaging techniques or by histological examination from tissue biopsies. Apart from the direct causes of it, NAFLD can also be caused by secondary causes such as the use of steatogenic medication for lengthy periods, in addition to genetic influences [5]. Liver steatosis—more scientifically known as hepatic steatosis—itself is considered a benign condition [6], but has several progressive stages, and if undiagnosed or untreated, may lead to liver cirrhosis, which is the most severe stage [2]. An intrahepatic triglycerides accumulation of at least 5% of liver weight or the presence of lipid vacuoles in 5% of hepatocytes without any secondary contributors is defined as hepatic steatosis [6].

Several factors are contributors to NAFLD. Overall, they can be classified as genetic, epigenetic, and environmental factors. Genetic predisposition, dietary habits, physical activity, and socioeconomic factors are among the main environmental factors [7]. Among these, the most common factors are those which are common for metabolic syndrome (MetS) as well, such as obesity, diabetes, and dyslipidemia—alone or in combination. Other factors also include age, gender, ethnicity, a history of fatty liver, and premature cardiovascular diseases [8].

A better understanding of the intertwined association of these factors, especially dietary factors, can be attained by following the pathogenesis of NAFLD. NAFLD is now considered to be caused by a multitude of conditions in which insulin resistance is the main factor that contributes to it, which in turn is caused by obesity. The above-mentioned factors cause excess fat and triglycerides to be deposited within the hepatocytes, and the lesser exiting of these into the bloodstream—resulting in NAFLD [9]. The relation between NAFLD and MetS is therefore considered bidirectional, as each of these leads to the other [10]. It is not just the insulin resistance, but rather the entire constellation of MetS which leads to excess fat deposition in the liver.

Dietary modifications are one of the methods of treatment of NAFLD. Several dietary approaches have been studied over the years, such as energy-restricted diets, diets rich in omega- 3 fatty acids, low glycemic index/load diets, diets with high total antioxidant capacity, moderate-high protein diets, high meal frequency patterns, and the Mediterranean diet (MD) [11]. The MD is considered to be one of the most recognized healthy and sustainable dietary patterns, and is characterized by the high consumption of plant-based foods such as fruits, vegetables, whole grains and legumes, seeds and nuts, and olive oil (as the major source of fats). However, MD includes a moderate intake of fish, a low-to-moderate intake of dairy products, a low intake of sweets and meat (especially red meat), and a moderate intake of alcohol (primarily in the form of wine that is consumed with meals) [12]. MD has been positively associated with numerous positive effects [13,14]. The first scientific evidence on the health benefits of the MD derives from the Seven Countries Study [15], in which Keys and colleagues documented a low incidence and mortality due to cardiovascular disease (CVD) among subjects living in the Mediterranean area compared to other countries. Successively, other observational studies have been performed that found a higher relationship of the MD with a reduction of cardiometabolic risk, diabetes, and certain cancers [16,17,18,19,20,21,22,23,24]. Recently, MD has also been considered as a potential dietary strategy in counteracting and/or mitigating NAFLD and related risk factors/disorders associated with this condition. The beneficial effects have been attributed to the numerous nutrients and non-nutrients present in the MD. The diet is high in mono-unsaturated fatty acids (MUFAs), polyunsaturated fatty acids (PUFAs), and fibers, which have been shown to have a beneficial effect on glucose and lipid metabolism and, consequently, on fatty liver disease [25,26,27]. MUFAs have been shown to improve waist circumference, high-density lipoprotein cholesterol (HDL-C), triglycerides (TGs), and glucose levels [28,29]. PUFAs, omega-3 fatty acids in particular, have been reported to improve insulin sensitivity and to reduce inflammation and oxidative stress [30,31]. Dietary fibers have been shown to exert cholesterol-lowering activity and positively modulate gut microbiota composition, which in turn increases the production of short-chain fatty acids with potential health benefits against NAFLD [32,33]. However, MD is also an important source of numerous bioactive compounds, such as (polyphenols (PPs). Several studies have hypothesized the contribution of PPs in the management of NAFDL thanks to their capacity to modulate the lipid metabolism and mitochondrial function, in addition to reducing inflammation and oxidative stress [34,35,36].

In the last decade, numerous dietary intervention studies have been performed to assess the benefits of MD in NAFLD subjects. In this systematic review, we investigated the effects of MD on NAFLD and related parameters by taking into consideration only randomized, controlled trials (RCTs). A meta-analysis was carried out to quantify the current evidence through the measurements of the standard anthropometrics, glycemic indices, liver function tests (LFTs), intrahepatic fat (IHF), fatty liver indices (FLI), and other biochemical parameters.

## 2. Materials and Methods

The protocol of the systematic review is registered in the International Prospective Register of Systematic Reviews (PROSPERO)—CRD42022367902.

### 2.1. Search Strategy

English-written articles, published from 2013 to 2023, were identified by searching the Google Scholar, PubMed, and Scopus databases. The search strategy on PubMed was based on the following MESH search terms (updated on 1st of March 2023): Non-alcoholic fatty liver disease (MeSH Terms) OR “Non-alcoholic Fatty Liver Disease” (MeSH Terms) OR NAFLD (MeSH Terms) OR hepatic steatosis (MeSH Terms) AND Mediterranean diet (MeSH Terms) OR Mediterranean-Diet (MeSH Terms) AND RCTs (MeSH Terms). A manual search was performed by two independents senior researchers with experience in clinical nutrition, through the revision of reviews and research articles on MD and metabolic syndrome. This search strategy (using the same terms) was also applied to Google Scholar and Scopus.

### 2.2. Study Selection

The selection process of the studies was based on PRISMA guidelines [37]. All randomized control trials conducted on humans within the last 13 years (from 2010–2022) were included. Non-English language studies, animal-based studies, in vitro studies, non-RCTs in overweight and obese patients, and RCTs in adults with BMI < 25 kg/m^2^ were excluded. Studies based on adolescent subjects, those not including a control group, and measured outcomes of interest other than that required were also excluded.

A more detailed list of criteria adopted is reported here. In particular, a structured approach using five components was adapted to construct the research question and to select the studies. The five components (PICOS) include (1) participants, (2) interventions, (3) comparators, (4) outcomes, and (5) study design.

#### 2.2.1. Participants

Adult participants (age ≥ 18 years) and overweight and obese adults (BMI ≥ 25 kg/m^2^) that were diagnosed with NAFLD were selected. No constraints were assigned concerning gender, disease, race, or the geographical distribution of the individuals enrolled in the study.

#### 2.2.2. Intervention

RCT investigated the effect of the MD on NAFLD by evaluating the IHF content, LFTs, NAFLD tests (Steatosis and fibrosis), and anthropometric and body composition assessments.

#### 2.2.3. Outcomes

Eligible studies were required to report baseline and follow-up values, the mean change (Δ-change) and relative standard deviation from baseline, and/or the mean differences among intervention groups vs. control groups concerning anthropometric outcomes such as body weight (BW), BMI, and waist circumference (WC).

#### 2.2.4. Study Design

Randomized controlled trials (RCTs) with the MD as the primary treatment and different control diets have been considered in patients with NAFLD.

### 2.3. Data Extraction and Analysis

Two authors (S.P. and A.R.) independently analyzed studies for their eligibility based on the following inclusion and exclusion criteria. Any disagreement between reviewers was resolved by consulting a third independent reviewer (C.D.B). For each study, the following data were collected: first author, publication year, study setting, study design, eligibility criteria, number of subjects, gender, age, race-country, intervention methods, treatment duration, and the main outcomes.

### 2.4. Risk of Bias in Individual Studies

Two authors from Bahrain independently assessed the risk of bias. Disagreements were solved by a third author (S.P.). The risk of bias in each study was assessed using the Cochrane Collaboration using the Risk of Bias tool [38] and considering factors contributing to the study quality, the generation of the allocation sequence, the allocation concealment, the blinding of outcome data, the presence of incomplete data, and selective reporting.

These factors were classified as having a low risk of bias, high risk of bias, or unclear risk of bias. Studies with a low risk of bias for at least three items were determined to be good, studies with a low risk of bias for at least two items were considered to be fair, and studies with a low risk of bias for no item or for only one item were regarded as poor.

### 2.5. Statistical Analysis

The study authors were contacted to gather the missing or unclear data. For continuous outcome data, the method used in the original study to account for missing data, usually the mixed model repeated measures or the last observation carried forward was used. The missing SD was calculated from *p*-values; to combine the two outcomes in our meta-analysis, the standardized mean difference (SMD) with 95% confidence intervals (CI) as the pooled effect size was used. Heterogeneity across the included studies was confirmed by using the Higgins’ *I*^2^ statistic. A fixed-effects model for data pooling was used if the *I*^2^ statistic was below 50%, which meant that there was acceptable heterogeneity across the included studies. The publication bias was checked through a meta-analysis or subgroup analysis including five or more studies. The level of significance was set at *p* < 0.05 for all statistical analyses performed. Procedures related to data pooling were carried out in Review Manager 5.4 software.

## 3. Results

### 3.1. Database Search

**Figure 1:**
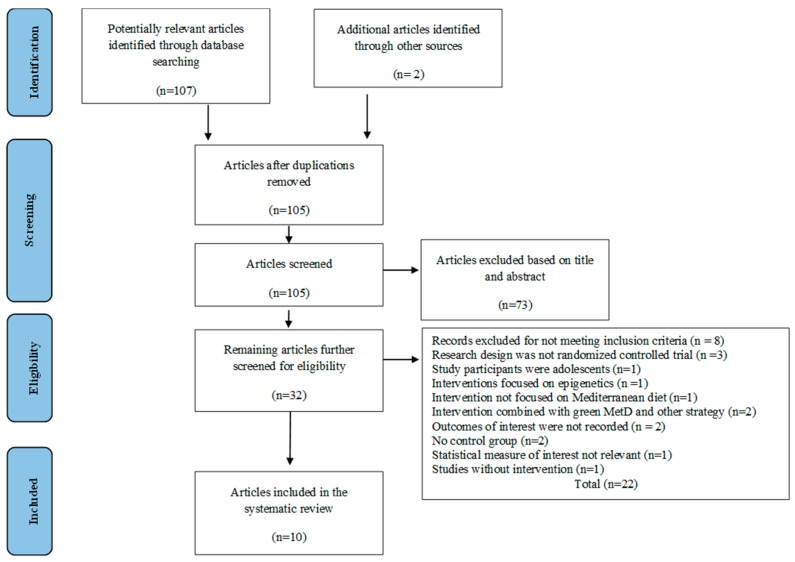
PRISMA flowchart of the study selection process.

The databases’ literature searches yielded a total of 107 potentially relevant studies. A total of 109 studies were found after reference networking of earlier systematic reviews revealed two additional pieces of research. After duplicates were eliminated, 105 studies remained. The initial filtering of titles and abstracts of the articles left 32 possibly suitable articles. Specifically, 22 articles were still excluded after the second round of eligibility screening, mostly because the research design was not a randomized control trial, the study participants were not adults, the intervention and outcome did not meet the inclusion criteria, the combined intervention did not follow the MD exactly, there was no parallel control group, or there was no intervention at all. As a result, 10 RCTs were chosen to be part of the current systematic review. Figure 1 shows the study selection procedure.

### 3.2. Study Characteristics

The studies that were chosen for this systematic review are listed in Table 1. Except for two studies involving populations from Australia, each study’s geographic origin was distinct, but they were all mostly from southeastern European countries including Serbia, Greece, Italy, and Spain. Additionally, several intervention techniques were used, with the chosen studies placing particular emphasis on energy restriction for the MD, increased energy expenditure, low SFA (saturated fatty acids), and high MUFA (monounsaturated fatty acids) and PUFA (polyunsaturated fatty acids). Regarding the study design, 10 papers were double-blind RCTs, the intervention lasted between one and twelve months, and the population’s age range was between 18 and 80 years. There was a total of 737 individuals in all from 10 studies, including both males and females (Table 1).

The control groups were set to consume either a hypocaloric conventional diet alone or with physical activity, or were given instructions about the range of macronutrient percentages to be consumed along with counseling. The intervention group received a hypocaloric dietary intake of 25–30% of the baseline intake, and increased energy expenditure by 400 kcal/70 kg of body weight. Macronutrients were set to be distributed based on the classic MD and/or Cretan diet, where carbohydrates accounted for up to 40–50% of the diet, fats for 30–40% of the diet, and proteins for 15–25% of the diet, with the reduced consumption of saturated fats (reducing to less than 10%), increased MUFAs and PUFAs (up to 22% and 9% of energy intake respectively), and increased fiber intake (approximately 25–30 g/day). The meal frequency was set to 5–7 meals per day, with reduced caloric content at each main meal, along with increased physical activity. The primary outcome of the studies was the assessment of the change in ALT, GGT, the blood lipid profile, liver function tests, and insulin resistance. Secondary outcomes included total cholesterol, liver fibrosis, and waist circumference.

#### 3.2.1. Anthropometric Variables

Out of the ten eligible studies, nine reported BMI, BW [39,40,41,42,43,44,45,46,47], and waist circumference (WC) [39,40,41,42,44,45,46,47,48]. Overall, 471 patients were assessed according to BMI and BW, while 539 were assessed according to WC. The study duration ranged from 1.5 months [46] to 12 months [39].

One hundred and twenty-eight patients were divided into two dietary intervention groups in a study by Montemayor et al., and Abbate et al.—the MD with high meal frequency for a period of 12 months [39,40]. In these prospective randomized trials, BMI, BW, and WC decreased substantially compared to the control group, which was given a conventional diet.

The effectiveness of the MD, together with energy restriction and seven 60-min counseling sessions (aimed at weight reduction and boosting adherence to MD), compared to a standard energy restriction regimen on NAFLD, was examined in an RCT done by Katsagoni et al. [42]. When compared to the control group, the MD group showed decreased BMI while having no noticeable changes in weight and WC. In the study of Properzi et al. [44] 51 NAFLD patients were divided into two dietary intervention groups (MD or low-fat (LF) diet) for three months. In this randomized parallel study, BW and BMI did not substantially change following the low-fat diet; however, the MD lowered WC in NAFLD patients. Three trials found that the MD reduced NAFLD patients’ BMI, weight, and WC. Ristic-Medic and coworkers [45] also depicted that all of the anthropometric parameters showed a significant improvement after a 12-week dietary intervention of MD or a low-fat diet on 27 NAFLD patients. Conversely, Ryan et al. [46] found a relatively small reduction in BW, while no change in WC and BMI was observed.

#### 3.2.2. Lipid Profile

Six out of the ten eligible studies reported the effect on the lipid profile [39,40,42,44,45]. Overall, 341 patients with NAFLD were assessed for triglycerides (TGs), total cholesterol (TC), high-density-lipoprotein-cholesterol (HDL-C), and low-density-lipoprotein-cholesterol (LDL-C) in these studies.

Montemayor et al. [39] and Abbate et al. [40] reported significant improvement in major biochemical lipid markers. An increase in HDL-C and a lowering of plasma triglycerides were significant post-interventions over a period of 6 and 12 months. However, no significant change in SBP, DBP, and LDL-C was reported by Abbate et al. [40]. In comparison to the control diet, Katsagoni et al. [42] discovered that MD can efficiently enhance HDL-C and decrease TC and LDL-C. Simultaneously, Properzi et al. [44] reported a significant reduction in TC and TGs post-3-month intervention with an energy-restricted, low-fat MD and counseling, while no significant increase in HDL-C and reduction in LDL-C was reported. Ristic-Medic et al. [45] also showed a significant reduction in TG, TC, and LDL-C following a low-fat MD for a duration of 3 months among a small population of 27 men. In comparison to the low-fat diet, the MD had no noticeable impact on LDL-C, HDL-C, TG, or TC according to an Australian study [46]. Abenavoli et al. [47] conducted a study to examine the effects of the MD in overweight patients with NAFLD, either with or without the addition of an antioxidant complex supplement. Three groups of patients were randomly assigned to each other: Group A had a low-calorie MD; Group B received a daily antioxidant supplement; and Group C received no treatment. According to the results, MD, either by itself or in combination with an antioxidant complex, significantly improved TC and TG when compared to the control group. However, the LDL-C and HDL-C did not show any significant increases.

#### 3.2.3. Glycemic Indices

Nine studies measured the effects of MD on HOMA-IR, insulin, and fasting glucose [40,41,42,43,44,45,46,47,48]. In total, 530 patients with NAFLD were assessed for their glycemia indices before and after intervention with MD. Abbate et al. [40] reported a reduction in HbA1c and HOMA-IR, while there was no significant reduction in fasting glucose among the MD, high meal frequency intervention group. While concurrent research by Katsagoni et al. [42] found that MD had no significant change in insulin, HOMA-IR, or fasting glucose following a six-month intervention, although the MD did lower HOMA-IR and insulin more than a low-fat/high-carbohydrate diet did. Similarly, Marin-Alejandre et al. [43], Ristic-Medic et al. [45], and Abenavoli et al. [47] showed statistically significant reductions in HOMA-IR after an intervention with an energy-restricted MD for a duration of 6 and 3 months, respectively. Franco et al. [48] also reported a reduction in HOMA-IR in all intervention arms. George et al. [41], Properzi et al. [44], and Ryan et al. [46], showed no significant reduction in glycaemic indices.

#### 3.2.4. Liver Enzymes

Nine out of the ten eligibly selected studies assessed the effect of MD on the major liver enzymes alanine aminotransferase (ALT), aspartate aminotransferase (AST), and gamma-glutamyl transferase (GGT) [39,40,41,42,43,44,45,46,47], meaning that a total of 472 patients with NAFLD were assessed.

Montemayor et al. [39], Abbate et al. [40], George et al. [41], Ryan et al. [46], and Abenavoli et al. [47], reported a reduction in ALT, AST, and GGT; however, the reduction was not statistically significant, except for GGT, in the study by Abenavoli et al. [47]. Conversely, Marin-Alejandre et al. [43] and Ristic-Medic et al. [45] reported a statistically significant reduction in all of these enzymes.

#### 3.2.5. NAFLD Severity Indices

All ten studies measured the severity of NAFLD using various indices. Seven measured the intrahepatic fat content (IHF) [39,40,41,42,43,44,46]; six measured hepatic stiffness and steatosis [39,40,42,43,45,47]; and three measured the fatty liver index (FLI) as a score to indicate severity [43,45,47]. Ryan et al. [46], Properzi et al. [44], Marin-Alejandre et al. [43], and Abbate et al. [40] reported statistically reduced IHF; whereas IHF was reduced but was not statistically significant, as reported by George et al. [41], and Montemayor et al. [39] following a MD intervention. Regarding FLI, Abenavoli et al. [47], Marin-Alejandre et al. [43], and Ristic-Medic et al. [45] reported a statistically significant reduction in FLI, rendering a positive impact of MD on NAFLD. A significant reduction in hepatic stiffness was only reported by Abenavoli et al. [47] and Ristic-Medic et al. [45].

### 3.3. Meta-Analysis of Randomized Control Trials

**Figure 2:**
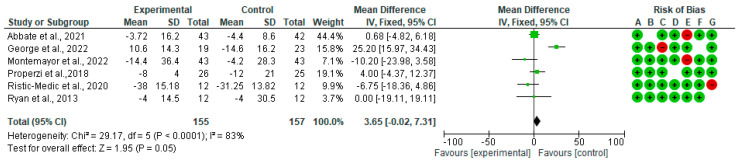
Effect of MD on ALT [39,40,41,44,45,46].

Figure 2 shows the forest plot for randomized controlled trials of MD studies included in an ALT (IU/L) subgroup meta-analysis (*n* = 270). The meta-analyzed data showed a not statistically significant decrease in ALT in the intervention group compared with the control diet.

**Figure 3:**
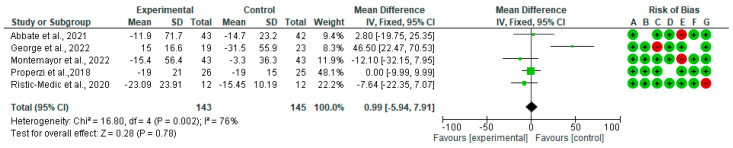
The effect of the MD on GGT [39,40,41,44,45].

Figure 3 shows the forest plots for randomized controlled trials of MD studies included in the GGT (IU/L) subgroup meta-analysis (*n* = 246). As shown in Figure 3, MD does not affect GGT (IU/L).

**Figure 4:**
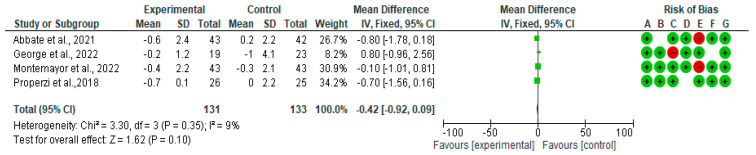
The effect of the MD on liver stiffness [39,40,41,44].

A forest plot for randomized controlled trials of MD studies included in the liver stiffness (kPa) subgroup meta-analysis (*n* = 222) is shown in Figure 4. As indicated, the MD decreased liver stiffness (kPa) by −0.42 (CI95% −0.92, 0.09) (*p* = 0.10). The test for overall effect was Z: 1.6 (*p* = 0.1). The heterogeneity was good at *I*^2^ = 9%.

**Figure 5:**
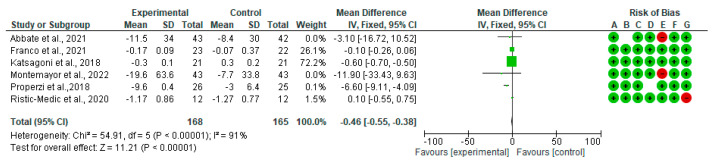
The effect of the MD on TC [39,40,42,44,45,48].

Forest plots for randomized controlled trials of MD studies included in the TC (mg/dL) subgroup meta-analysis (*n* = 333) is reported in Figure 5. The meta-analysis has shown that MD significantly affects TC. The mean difference in TC across all the studies was −0.46 mg/dL (CI95% −0.55, −0.38) (*p* = 0.00001). The test for overall effect was Z: 11.21 (*p* = 0.0001). The heterogeneity was *I*^2^ = 91%.

**Figure 6:**
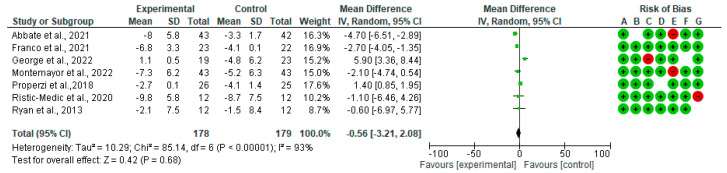
The effect of the MD on WC. Abbreviations: SD: standard deviation; mean: mean difference changes pre-post [39,40,41,44,45,46,48].

In Figure 6, we show the forest plots for randomized controlled trials of MD studies included in the WC (mg/dL) subgroup meta-analysis (*n* = 357). The meta-analysis showed that MD affects the WC. The mean difference in WC across all the studies was −0.56 cm (CI95% −3.21, −2.08) (*p* = 0.69). The heterogeneity was *I*^2^ = 93%.

### 3.4. Risk of Bias

**Figure 7:**
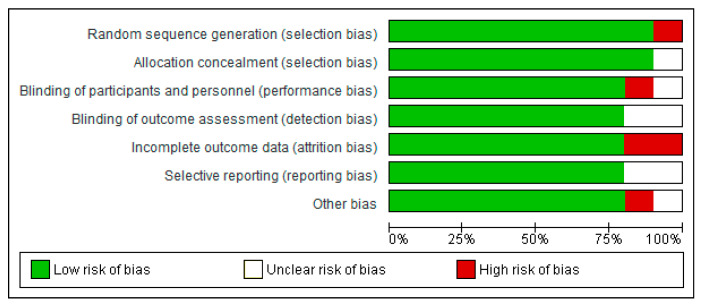
Risk of bias summary.

All trials were classed as having a low risk or an unclear risk of bias based on one or more of the components. Overall, over 75% of the selected studies showed a low risk of bias (Figure 7).

## 4. Discussion

The following systematic review and meta-analysis revealed that MD has desirable effects on NAFLD subjects by significantly reducing the TC and liver stiffness. Although the MD has shown a tendency to improve the liver enzyme profile, lipid profile, and WC, the differences were not significant.

The beneficial findings observed at the hepatic level following intervention with the MD were consistent with those found by Kawaguchi et al. [49] in their meta-analysis, where an MD intervention significantly reduced liver stiffness. A comparison of the various hepatological parameters indicated a reduction of liver fibrosis of between 0.5 and 2.1 kPa. It has also been shown that there was a significant relationship between improving the contents of fat in the liver and following a MD. Among the many health benefits are a reduction in the symptoms of metabolic syndrome, the improvement and reduction in the symptoms of diabetes, and in the reversing of fatty liver disease [50,51,52,53]. The use of the MD affects liver enzymes by improving proteins associated with liver functions in general, and the most interesting finding was that the MD significantly reduced AST, but had no significant effect on ALT [52]. An inverse relationship between MD and NAFLD was reported by Baratta et al. [52], where they also found a significant reduction of hepatic steatosis following MD intervention. A comparison of groups based on their adherence to MD showed not only a significant reduction in insulin levels, but also found a 36% lower chance of hepatic steatosis among those adhering to a MD [52]. In the meta-analysis of Haigh et al. [54] the authors showed that MD induced a reduction in ALT activity and liver stiffness in subjects with NAFLD. However, the effect of MD deserves more investigation, since other studies reported conflicting findings. For example, Moosavian et al. [55] found that five out of eight studies included in their meta-analysis did not show any significant effects on liver enzymes.

The role of MD in the modulation of lipid profiles has been evaluated in numerous systematic reviews and meta-analyses [20,56,57,58,59]. For example, Uli et al. [57], in their systematic review, showed that MD could lower LDL, TGs, TC, and fasting blood glucose levels, while increasing HDL-C in overweight and obese individuals. A recent review and meta-analysis of 13 RCTs analyzed the effectiveness of the MD versus a conventional low-fat diet on several metabolic outcomes, including markers of lipid profile, in subjects at high-risk living in non-Mediterranean countries. The results revealed that MD was only significantly superior to the low-fat diet in reducing TC, which is in line with our findings, while the not significant effect was documented for the rest of the markers of the lipid profile [58]. Dinu and coworkers [20], in their umbrella review of a meta-analysis of RCT (performed in the different target populations), reported that the beneficial effects of MD were mostly related to anthropometric parameters and cardiometabolic risk factors, in particular TC. Conversely, Neuenschwander et al. [59], in their systematic review and network meta-analysis of studies performed in subjects with type 2 diabetes mellitus, reported a positive effect on HDL-C and TG levels, but not TC, compared to the control diet. This discrepancy between findings could be attributed to the different target populations considered. Regarding NAFLD, very few meta-analyses have been carried out. Asbaghi and colleagues [60] reported that a MD significantly decreased the serum levels of TG and TC (other than HOMA-IR) in comparison to a control diet in NAFLD patients. Moosavian et al. [55] documented an improvement in the lipid profiles and other NAFLD severity indices among patients with NAFLD, but differences between studies highlight the need for more clinical trials with adequate sample sizes and better methodologies.

The administration and following-up of the MD are beneficial as a weight loss strategy among overweight and obese people [61]. Prior studies reported that subjects with high adherence to the MD showed greater decreases in BMI and BW [62]. A systematic review and meta-analysis on adherence to MD showed a significant reduction in WC [63]. Other meta-analyses reported a beneficial effect of MD in the control of BW, WC, and other anthropometric parameters in subjects with NAFLD [55,61].

Energy-restricted MD seems to be a wise choice for people at a high risk of cardiovascular disease given the health advantages and compatibility with weight loss. However, Thom et al. [64] reported that, regardless of the type of diet and macronutrient composition, weight reduction improves almost all metabolic markers, including cardiovascular disease. They found similar results in terms of weight reduction when compared to low-fat and low-carbohydrate diets [65]. Another RCT found MD to be a good dietary approach to reducing body fat mass [66].

### 4.1. Potential Mechanisms

Several synergistic interactions among food components could explain the beneficial effects of MD on NAFLD. MD is characterized by a low intake of lipids (since mostly plant-based) and the SFAs. Several studies have shown that a reduced intake of SFAs is associated with a reduction in TC, LDL-C, and TGs [67,68]. SFA may promote cellular dysfunction by activating ER stress pathways, upregulating NAFLD-associated pathways, and both systemic and hepatic insulin resistance, as well as contributing to the intrahepatic triglyceride (IHTG) pool [69,70]. It was initially believed that dietary fats constituted the smallest source of lipids that could enter the IHTG pool, contributing roughly 10% to 20% of liver TG fatty acids compared with the plasma free fatty acid (FFA) pool (from 60% to 100% of liver TG fatty acids), and with de novo lipogenesis (up to 30% to 40% of IHTG) [70,71]. However, a recent study by Lindeboom et al. [72] showed the incorporation of 13C-labeled fatty acids into IHTGs after a single high-fat meal, suggesting that a substantial amount of liver fat can be derived directly from the storage of meal-derived fat, particularly given the increased insulin levels that would be expected following such a meal. While increased hepatic TG formation represents an early indicator of liver metabolic stress and disease, it does not appear to be the initiating factor in nonalcoholic steatohepatitis, but instead, TG may serve as inert storage and as a protective metabolic mechanism to counter FFA overload and reduce potential lipo-apoptotic effects.

MUFAs represent the main source of fatty acids in MD. It has been proved that MUFA intake may prevent the development of NAFLD by improving plasma lipid levels, reducing body fat accumulation, and decreasing postprandial adiponectin expression [73]. The beneficial effects of MUFA in the context of NAFLD were also observed in our review, which showed that the intake of MUFA up to 22% of the total daily energy intake lowered the TC. In fact, the meta-analysis showed consistency with a significant reduction in TC following the MD, which is in line with the observations reported in other studies. Olive oil represents the main MUFA source of the MD, and it could in part explain the findings obtained. Numerous studies have reported the beneficial effects of olive oil in reducing cardiovascular risk, improving lipid metabolism (preventing the oxidation of LDL-C, thereby reducing LDL atherogenesis), and glycemic levels [74,75]. Other studies documented an increase in the HDL-mediated macrophage cholesterol efflux capability, HDL antioxidant activity, and HDL anti-inflammatory features [76]. In addition, a significant improvement in blood pressure and endothelial function among hypertensive patients was observed [77,78].

PUFAs are an important component of MD. They are involved in the control of three crucial transcriptional factors controlling the hepatic carbohydrate and lipid metabolism. PUFAs activate hepatic peroxisome proliferator-activated alpha, thus enhancing FA oxidation. PUFAs induce the suppression of sterol regulatory element binding protein-1 and of carbohydrate regulatory element binding protein/Max-like factor X, resulting in the inhibition of glycolysis and de novo lipogenesis. In addition, PUFAs promote a shift in metabolism from FA synthesis and storage toward FA oxidation, with a favorable effect on hepatic steatosis [79]. Furthermore, PUFAs may act by controlling the inflammation process occurring in non-alcoholic steatohepatitis (NASH) [80,81], with an opposite effect found for omega 3 and omega 6. Specifically, omega-3 may improve and induce an independent anti-inflammatory effect via the suppression of tumor necrosis factor (TNF) and interleukin-6 (IL-6), which are mainly involved in the inflammation process [82,83], while omega-6 may have a pro-inflammatory role due to their direct relationship with the production of arachidonic acid and thus to the eicosanoids as pro-inflammatory mediators [80].

MD can also contribute to lowering plasma cholesterol through the intake of fiber, in particular water-soluble fibers that are found in large concentrations in some MD foods, mainly beans, vegetables and fruits, and whole-grain cereals. Water-soluble fibers have been shown to increase the rate of bile excretion, thereby reducing total serum and LDL cholesterol [82]. This association has also been proven by Zhao et al. [82], who showed a statistically significant negative association between the intakes of total cereal, fruit, and vegetable fiber with NAFLD among over 6000 participants over 20 years of age. Furthermore, these findings are backed up by recent findings, in which participants diagnosed with NAFLD and having clinically significant fibrosis (CSF), showed lower odds of NAFLD and CSF among those consuming higher fiber diets compared to those consuming less fiber [83]. The benefits of dietary fiber have also highlighted the potential role the gut microbiota plays in the improvement of NAFLD, weight loss, as well as in the improvement of metabolic diseases, in that the higher consumption of dietary fiber (mainly in the form of oligofructose), decreases the gut dysbiosis by providing beneficial microbes such as *Bifidobacteria* and improves gut permeability [84].

MD is also characterized by the high content of numerous bioactive compounds such as carotenoids [85,86], but of PPs above all others [87,88]. PPs are secondary metabolic products of plants, and for this reason are widely distributed in fruits, vegetables, whole grains, and olive oil [88]. Recent studies documented the potential role of PPs, and related subclasses, in the modulation of NAFLD, even if the real mechanism has yet to be determined [89,90]. Several independent mechanisms have been identified and can, at least in part, explain the prevention of the progression of liver damage [36,90,91]. The first is the inhibition of lipogenesis by reducing SREBP-1c, which is recognized as being the target gene involved in lipid biosynthesis in the liver. PPs, such as anthocyanins, seem to act by reducing de novo lipogenesis through the down-regulation of SREBP-1c [35]. The second is the promotion of lipolysis by increasing β-FA oxidation via PPARα upregulation. Both the down-regulation of SREBP-1c and the upregulation of PPARα seem to be modulated by AMPK activation (by phosphorylation) at the hepatic level. The third mechanism involves improving insulin sensitivity. Postprandial insulin secretion promotes hepatic glucose uptake and glycogen synthesis by inhibiting gluconeogenesis. Most studies are in agreement with the fact that a range of PPs reduces hepatocellular TG accumulation induced not only by fats, but also by high glucose concentrations [92,93,94,95]. For example, the epigallocatechin-3-gallate supplement has been shown to improve insulin sensitivity and promote the functional recovery of insulin receptor substrate-1 in a model of nonalcoholic steatohepatitis [95]. The fourth mechanism involves the reduction in oxidative stress and inflammation. Reactive oxygen species (ROS) appear to be significantly involved in a cascade of oxidative events that lead to hepatic damage and NAFLD progression. ROS triggers lipid peroxidation, in particular that of PUFAs, along with the formation of highly reactive aldehyde products such as malondialdehyde (MDA) and 4-hydroxy-2-non-enal (4-HNE) [96]. Furthermore, oxidative stress can, directly and indirectly, contribute to the up-regulation of the nuclear factor kappa-light-chain-enhancer of activated B cells (NF-kB) and pro-inflammatory cytokines (TNF-α, IL-6, and IL-1) that are involved in the apoptosis and development of hepatic fibrosis. PPs are recognized as being a strong inducer of nuclear factor-erythroid 2-related factor 2 (Nrf2), and consequently are involved in the production of numerous antioxidant enzymes that are able to counteract oxidative stress [97]. Furthermore, PPPs are known to attenuate inflammatory pathways thanks to their capacity to inactivate NF-κB [98].

### 4.2. Strengths and Limitations

The incorporation of RCTs for the systematic review and meta-analysis was the main strength of the study. However, this review is not short of limitations. It is likely that some studies included did not follow the exact MD macronutrient distribution due to the different variations of the MD administered (e.g., low-glycemic index, caloric restriction, Cretan diet). In addition, since the studies included were carried out in different countries (e.g., Italy, Greece, Serbia, Spain and Australia), the type of foods consumed was different. However, this information was not always available. Some studies provided food to the volunteers (e.g., extra virgin olive oil, nuts, canned fish and legumes), while others only provided dietary advice with the list of foods that were allowed. Furthermore, the geographical locations of the studies may also have affected the analysis in that some studies among those selected were on populations that normally do not follow a MD and lifestyle, such as those from Australia and Serbia. All of these aspects could have also have affected the overall results obtained. Another limitation may be the participants’ low adherence to the MD. Not all studies measured this adherence. Some measured the quality of life or secondary markers, such as weight loss or improved anthropometric measures, which are not the most accurate measures of adherence to a MD. With the lack of adherence in these studies, the risk of inaccuracy increases further. Participants’ responses in some studies involved self-reported methods in answering the food-frequency questionnaire (FFQ), which may have introduced other sources of errors such as recall bias. Other than these, using non-invasive techniques for measuring liver fat content, which is less accurate than the invasive method of biopsy, has limitations. The small study sample size was another limitation of the studies involved in this review. Larger study samples with longer intervention periods may provide even better results.

## 5. Conclusions

The comprehensive summary of the effect of MD on NAFLD showed the great potential of this dietary pattern in improving the parameters associated with NAFLD severity, such as improving the liver function enzymes and the NAFLD scores. Along with this, MD reduced the waist circumference and some liver enzymes; however, the reduction was not significant. The meta-analysis results show a statistically significant reduction in total cholesterol and liver stiffness.

Further research is needed to obtain enough data on larger populations and in different countries to reach an even more objective and extensive answer to our question.

## Figures and Tables

**Table 1:** Characteristics of the studies.

First Author/Year of Publication	Sample, Gender (Control, Intervention Groups)	Country	Population (Age, BMI)	Treatment Duration	Intervention Strategies
Montemayor et al. [39]	Total: 128 (128 F) CD:43 MD-HMF:43	Spain	NAFLD patients with MetS of 40–60 years of age and BMI 27–40 kg/m^2^	12 months	Energy restriction MD, and increased energy expenditure
Abbate et al. [40]	Total: 128 (128 F) CD:43 MD-HMF:43	Spain	NAFLD patients with MetS of 40–60 years of age and BMI 27–40 kg/m^2^	6 months	Energy restriction MD, and increased energy expenditure
George et al. [41]	Total: 42	Australia	NAFLD patients of over 18 years of age and BMI of 32 ± 6 kg/m^2^	3 months	Low-fat diet, MD
Katsagoni et al. [42]	Total: 63 Control: 21 MD group: 21	Greece	NAFLD patients of 18–65 years of age and BMI 25–40 kg/m^2^	6 months	Energy restriction MD, counseling, increased energy expenditure
Marin-Alejandre et al. [43]	Total: 76 AHA (control) = 37 FLiO = 39	Spain	NAFLD patients of 40–80 years of age and BMI of 27.5–40 kg/m^2^	6 months	Energy restriction, MD
Properzi et al. [44]	Total: 51	Australia	NAFLD patients who are also overweight	3 months	Energy restriction and the low-fat MD
Ristic-Medic et al. [45]	Total: 27 (only M)	Serbia	NAFLD patients who are also overweight	3 months	Energy restriction diet, low-fat or MD, counseling
Ryan et al. [46]	Total: 12 6 F, 6 M	Australia	NAFLD patients with MetS	1.5 months	Energy restriction MD, increased PUFA and MUFA
Abenavoli et al. [47]	Total: 50	Italy	NAFLD patients of 18–65 years of age and BMI over 25 kg/m^2^	6 months	Energy restriction MD; antioxidant supplementation
Franco et al. [48]	Total: 144 89 M, 55 F	Italy	Moderate to severe NAFLD patients of 18–65 years of age and BMI of 25–40 kg/m^2^	3 months	LGIMD—low saturated fats, high MUFA and PUFA intake, increased energy expenditure

Legend: F: females; M: males; CD: Control diet; MD: Mediterranean Diet; HMF: High Meal Frequency; AHA: American Heart Association; FLiO: Fatty Liver in Obesity; NAFLD: Non-alcoholic Fatty Liver Disease; MetS: Metabolic Syndrome; MUFA: Monounsaturated Fatty Acids; PUFA: Polyunsaturated Fatty Acids; GI: Glycemic Index; BMI: Body Mass Index; LGIMD: Low Glycemic Index Mediterranean Diet.

## Data Availability

Not applicable.
